# Recipe for pituitary organoids

**DOI:** 10.3389/fendo.2022.1025825

**Published:** 2023-01-19

**Authors:** Mayuko Kano, Hiroo Sasaki, Tsutomu Miwata, Hidetaka Suga

**Affiliations:** ^1^ Division of Metabolism and Endocrinology, Department of Internal Medicine, St. Marianna University School of Medicine, Kanagawa, Japan; ^2^ Department of Neurosurgery, Nagoya University Graduate School of Medicine, Nagoya, Japan; ^3^ Department of Endocrinology and Diabetes, Nagoya University Graduate School of Medicine, Nagoya, Japan

**Keywords:** corticotroph, embryonic stem cells, induced pluripotent stem cells, organoid, pituitary, pluripotent stem cells

## Abstract

Generation of a variety of organs and tissues from human pluripotent stem cells (hPSCs) has been attempted *in vitro*. We here present a simple and efficient method for induction of hypothalamic and pituitary tissues from hPSCs. On provision of exogenous agents important for early hypothalamus-pituitary organogenesis, including bone morphogenetic protein 4 and activators of sonic hedgehog, in three-dimensional culture, hPSCs spontaneously form spherical organoids with two distinct tissues, hypothalamus and adenohypophysis. The pituitary tissues derived from hPSCs not only secrete adenocorticotropic hormone, but also retain both positive and negative feedback mechanisms, recapitulating mature endocrine organs *in vivo*. Furthermore, the results of ectopic transplantation with mouse models of hypopituitarism suggest that these hypothalamus-pituitary organoids have potential as engraftment organs. In addition to their use in transplantation for patients with hypopituitarism they will allow establishment of disease models *in vitro* and enable research impossible in humans. Hypothalamus-pituitary organoids promise to be a powerful tool in regenerative medicine, drug discovery, and basic research into pituitary development.

## Introduction

**Figure 1:**
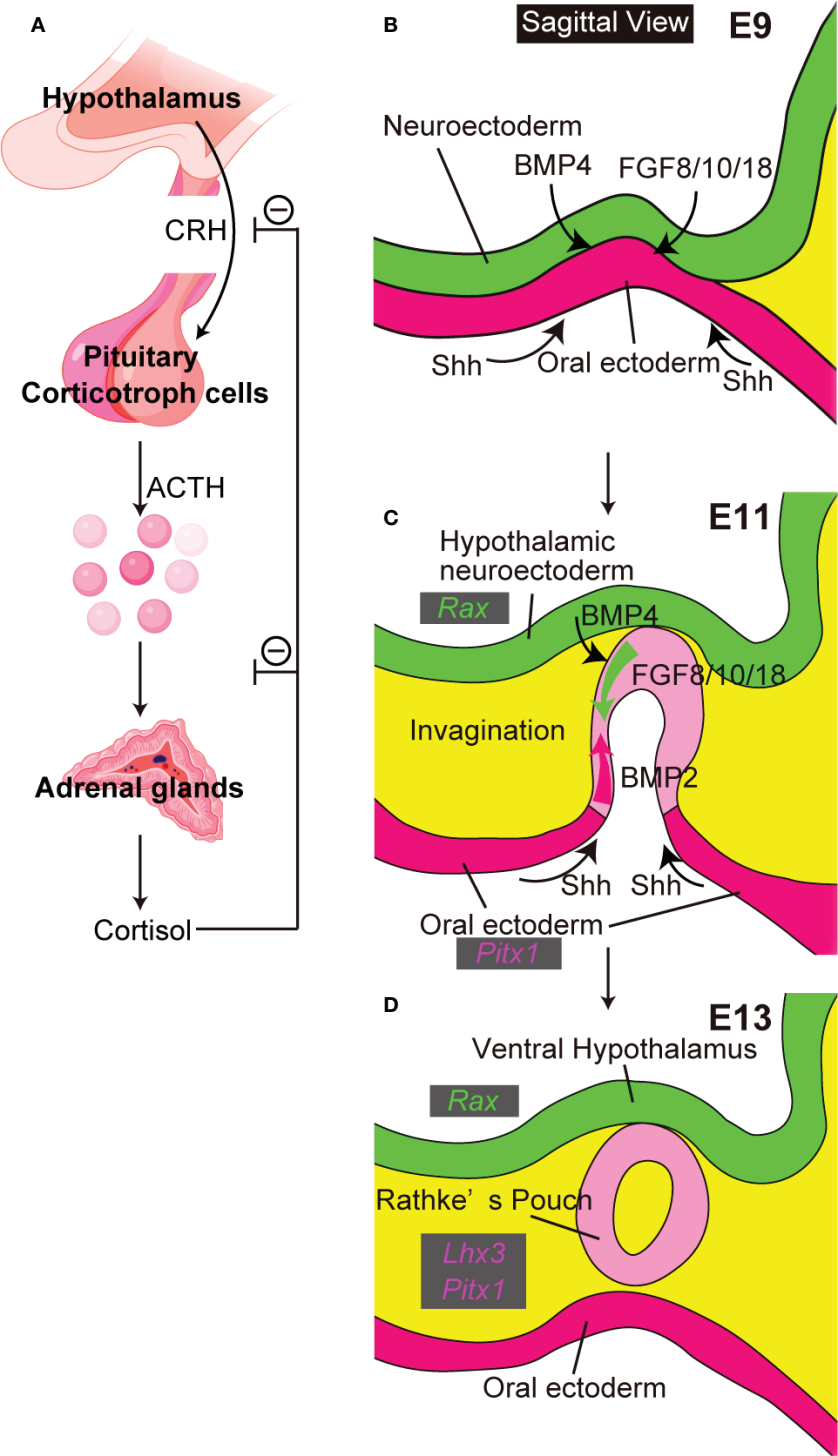
The close relationship between hypothalamus and pituitary. **(A)** The hypothalamus-pituitary-adrenal axis. Created with BioRender (https://biorender.com). **(B–D)** Pituitary organogenesis in mice. The ventral diencephalon provides BMP4 and FGF 8/10/18. The oral ectoderm provides signals that activate sonic hedgehog (Shh) **(B)**. Opposing dorsal→ventral FGF8/10/18 and ventral←dorsal BMP2 gradients are crucial for pituitary organogenesis **(C)**. Ventral hypothalamus expresses *Rax*. Non-neural oral ectoderm expresses *Paired like homeodomain 1* (*Pitx1*). *LIM homeobox protein 3* (*Lhx3*) encodes an early pituitary marker.

The hypothalamic-pituitary system coordinates endocrine functions essential for survival. Hypothalamic hormones secreted into the hypothalamic portal system directly activate anterior pituitary cell surface receptors. Differentiated adenohypophyseal cells secrete the six hormones adenocorticotropic hormone (ACTH, from corticotroph cells), growth hormone (GH, from somatotroph cells), prolactin (PRL, from lactotroph cells), thyrotropin-releasing hormone (TSH, from thyrotroph cells), and follicle-stimulating hormone (FSH) and luteinizing hormone (LH), both from gonadotroph cells. The hypothalamus-pituitary target gland axis is critical in regulation of pituitary hormone secretion and maintenance of homeostasis. The hypothalamus-pituitary-adrenal axis is well-known (**Figure 1A**). Corticotropin-releasing hormone (CRH) from the hypothalamus induces ACTH secretion from pituitary corticotroph cells. ACTH then stimulates the adrenal glands to secrete cortisol, which inhibits release of CRH and ACTH in an example of negative feedback control.

Pituitary insufficiency may be congenital or acquired. Congenital causes include developmental disorders and mutations in pituitary-related genes. In acquired pituitary insufficiency, pituitary function is rarely restored once lost. Thus patients with either form of hypopituitarism require long-term replacement therapy. In ACTH deficiency, patients receive glucocorticoid replacement therapy to avoid life-threatening acute adrenal insufficiency (adrenal crisis). Conventional replacement therapy aims to mimic normal cortisol secretion; however, its benefits are limited. Patients with adrenal insufficiency are at increased mortality risk that care using contemporary protocols does not normalize (1). The incidence of adrenal crisis in chronic adrenal insufficiency is high even in well-educated patients, demonstrating that conventional regimens do not satisfy the glucocorticoid demands of patients with chronic adrenal insufficiency who encounter illness or other stress (*e.g*., trauma, surgery requiring general anesthesia, and childbirth) (2). Perhaps to produce and to engraft differentiated pituitary cells from patients’ own induced pluripotent stem cells (iPSCs) could overcome clinical problems in current replacement therapy.

Efficient differentiation of telencephalic precursors from mouse embryonic stem cells (mESCs) was first achieved by serum-free, floating culture of embryoid-body – like aggregates (SFEB culture) (3). In SFEB culture, mESCs are dissociated into single cells and floating-cultured in serum-free medium containing knockout serum replacement (KSR). Dissociated mESCs spontaneously form floating aggregates within one day. SFEB-cultured mESCs differentiate into neural progenitors and efficiently generate brain factor 1 – expressing telencephalic progenitors. Modified SFEB culture (SFEBq) causes mESCs to differentiate into rostral hypothalamic progenitors (4). SFEBq differs from SFEB first in that dissociated mESCs quickly reaggregate in a low cell-adhesion culture well and second in that mESCs are cultured in a strictly chemically defined medium free of KSR and other growth factors including insulin (growth factor-free chemically defined medium, gfCDM). In about 25 days of SFEBq culture, mESCs differentiate into mature hypothalamic neurons *via* hypothalamic progenitor cells that express *Retina and anterior neural fold homeobox* (*Rax*).

Initial pituitary organogenesis begins around embryonic day (E) 8.5 in mice (**Figures 1B–D**). A part of the oral ectoderm adjacent to the midline ventral diencephalon thickens and invaginates toward the ventral diencephalon to form the future Rathke’s pouch. Various signaling molecules are involved in early pituitary development (5). Signals from both ventral diencephalon and oral ectoderm are crucial for development of Rathke’s pouch. The ventral-diencephalon signaling molecules include bone morphogenetic protein (BMP) 4 and fibroblast growth factor (FGF) 8/10/18. In contrast, the oral ectoderm provides a ventral sonic hedgehog (Shh) signal that induces BMP2 expression in the ventral pouch. Opposing dorsal→ventral FGF8/10/18 and ventral←dorsal BMP2 gradients appear key for pituitary organogenesis and terminal differentiation of pituitary cell types. These developmental findings encouraged attempts to differentiate mESCs into hypothalamus-pituitary units *in vitro* by recapitulating pituitary organogenesis. Mouse ESCs differentiated into pituitary progenitor cells that expressed the Rathke’s pouch marker Lim3 and in SFEBq culture, when treated with the sonic-hedgehog activator smoothened-agonist (SAG), formed pouch-like structures (6). On treatment with the Notch inhibitor (2S)-N-[N-(3,5-difluorophenacetyl)-L-alanyl]-2-phenylglycine tert-butylester (DAPT) these SAG-treated SFEBq aggregates further differentiated to include ACTH-producing corticotroph cells, with hypothalamic elements inside the organoids and pituitary elements at the organoids’ surfaces. Finally, on implantation beneath the renal capsule these mESC-derived pituitary tissues rescued the phenotypes of hypopituitary mice.

**Figure 2:**
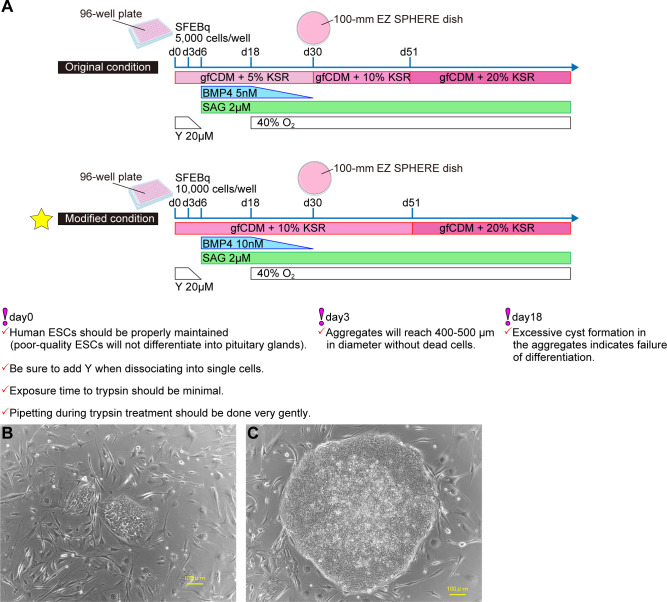
Efficient pituitary differentiation from hPSCs. **(A)** Pituitary differentiation from hPSCs and critical points for succesful induction. Above, original conditions (7). Below, modified conditions (8). This article describes the modified method. **(B)** Human PSCs one day after passage. **(C)** Human PSCs three days after passage. Healthy hPSCs have well-defined colony edges and high cell density in the colony center. Suitable colony diameter for passage is 1mm–2mm. Scale bars: 100 µm **(B, C)**.

Working from culture conditions in mESCs, pituitary differentiation from human ESCs (hESCs) has been achieved (7) (**Figure 2A**). SFEBq aggregates derived from hESCs were differentiated into Lim3-expressing pituitary progenitor cells by adding both SAG and BMP4. Unlike in mESCs, supplementation of not only SAG but also BMP4 is essential for differentiation of pituitary glands from hESCs. Moreover, to induce pituitary differentiation (ACTH-expressing cells) from mESCs requires ~ 21 days, while it takes ~ 60–100 days with hESCs. We established a protocol to improve the efficiency of pituitary differentiation from human iPSCs (8) (**Figure 2A**). Modified conditions substantially increased the proportion of ACTH-expressing cells and ACTH concentrations in culture medium. Some researchers have induced anterior pituitary cells to develop from hPSCs in two-dimensional (2D) adherent culture (9, 10). In contrast, the internal hypothalamus and the superficial pituitary coexist and differentiate together in our three-dimensional SFEBq aggregates. We believe that the relationship between hypothalamus and pituitary, paralleling that in hypothalamus-pituitary organogenesis *in vivo*, is critical for pituitary maturation *in vitro*.

Our studies have provided proof-of-concept (POC) for ectopic transplantation of hPSC-derived pituitary tissues as a treatment of hypopituitarism (7). Moreover, hPSC-derived hypothalamus-pituitary organoids are a powerful research tool, permitting insights into pituitary embryology and the pathogenesis of hereditary pituitary dysplasia. For example, pituitary organoids differentiated from patients-specific iPSCs have revealed that the product of *orthodenticle homeobox 2*, promoting pituitary progenitor cell differentiation, is implicated in the pathogenesis of congenital pituitary hypoplasia (11, 12).

This essay presents a highly efficient method of inducing pituitary differentiation from hPSCs (8) in which hPSCs differentiate over 60–100 days *via* oral ectoderm and pituitary progenitor cells into corticotroph cells that produce ACTH which can be measured in culture medium. Corticotroph cells derived from hPSCs release ACTH in response to CRH *in vitro* and *in vivo* and on transplantation beneath the renal capsule rescue the phenotype of SCID mice subjected to hypophysectomy (7). This differentiation protocol is uncomplicated; we believe that it is reproducible for anyone familiar with basic cell-culture techniques. We supply step-by-step instructions for making pituitary organoids “in a dish” and highlight key points for avoiding failure.

## Reagents

- Trypsin powder (Difco, cat. no. 215240)
- 4′,6-diamidino-2-phenylindole (DAPI) (Dojindo, cat. no. D523)
- Fetal bovine serum (FBS) (Equitech-Bio, cat. no. SFBM30-2537)
- Chemically defined lipid concentrate (CDLC) (Gibco, cat. no. 11905-031)
- Collagenase IV (Gibco, cat. no. 17104-019)
- L-glutamine (Gibco, cat. no. 25030-081)
- Non-essential amino acids (NEAA) (Gibco, cat. no. 11140-050)
- TrypLE Express (Gibco, cat. no. 12605-010)
- 0.05% trypsin – ethylene diamine tetraacetic acid (EDTA) (Gibco, cat. no. 25300-054)
- KnockOut serum replacement (KSR) (Invitrogen, cat. no. 10828-028)
- Mitomycin C 10 mg (Kyowa Kirin, cat. no. 4231400D1031)
- ACTH ELISA kit (MD Biosciences, cat. no. M046006)
- Butorphanol (Meiji Seika)
- 10 × Dulbecco’s phosphate-buffered saline (D-PBS) (-) (Nacalai Tesque, cat. no. 11482-15)
- DNase I (Roche, cat. no. 11284932001)
- Optimal cutting temperature (OCT) embedding compound (Sakura Finetek, cat. no. 4583)
- Midazolam (Sandoz, cat. no. 1124401A1060)
- Bovine serum albumin (Sigma, cat. no. A3156)
- CaCl_2_ (Sigma, cat. no. 05-0570)
- Dimethyl sulfoxide (DMSO) (Sigma, cat. no. D2650)
- Dulbecco′s modified Eagle′s medium (DMEM) (Sigma, cat. no. D5796)
- Dulbecco′s modified Eagle′s medium/nutrient mixture F-12 Ham (DMEM/F12) (Sigma, cat. no. D6421)
- 1-thioglycerol (Sigma, cat. no. M6145)
- Human CRH (Tanabe, cat. no. 7223406D1030)
- Isoflurane (Wako, cat. no. 9906571)
- 2-mercaptoethanol (2-ME) (Wako, cat. no. 131-14572)
- 4% paraformaldehyde (Wako, cat. no. 163-20145)
- Recombinant human basic fibroblast growth factor (bFGF) (Wako, cat. no. 060-04544)
- Sterile water (Wako, cat. no. 039-24155)
- Sucrose (Wako, cat. no. 196-00015)
- Triton X-100 (Wako, cat. no. 168-11805)
- Trypan blue solution (Wako, cat. no. 207-17081)
- Tween 20 (Wako, cat. no. 167-11515)
- Y-27632 (Wako, cat. no. 034-24024)
- Atipamezole (Zenoaq)
- Medetomidine (Zenoaq)

### Primary antibodies

Anti-RX, -LHX3, and -PITX1 are antibodies custom-produced in our laboratory and not commercially available.

Anti-ACTH (Fitzgerald, cat. no. 10C-CR1096M1, RRID: AB_1282437)

Anti-pan-cytokeratin (Sigma, cat. no. C2562, RRID: AB_476839)

Anti-E-cadherin (Takara, cat. no. M108)

Anti-Nkx2.1 (Zymed, cat. no. 18-0221, RRID: AB_86728)

### Secondary antibodies

Cy3 donkey anti-guinea pig (Jackson, cat. no. 706-165-148, RRID: AB_2340460)

488 donkey anti-mouse (Thermo Fisher Scientific, cat. no. A21202, RRID: AB_141607)

488 goat anti-guinea pig (Thermo Fisher Scientific, cat. no. A11073, RRID: AB_2534117)

546 donkey anti-goat (Thermo Fisher Scientific, cat. no. A11056, RRID: AB_2534103)

546 donkey anti-rabbit (Thermo Fisher Scientific, cat. no. A10040, RRID: AB_2534016)

546 donkey anti-mouse (Thermo Fisher Scientific, cat. no. A10036, RRID: AB_2534012)

546 goat anti-chicken (Thermo Fisher Scientific, cat. no. A11040; RRID, AB_2534097)

546 goat anti-guinea pig (Thermo Fisher Scientific, cat. no. A11074, RRID: AB_2534118)

647 goat anti-rat (Thermo Fisher Scientific, cat. no. A21247, RRID: AB_141778)

## Cells

### 1 Mouse embryonic feeder cells (MEF feeder cells)

MEF, Kitayama Labes, KBL9284600

### 2 Human ESCs

KhES-1, RIKEN BRC, HES0001

KhES-3, RIKEN BRC, HES0003

### 3 Human iPSCs

201B7, RIKEN BRC, RBRC-HPS0063

409B2, RIKEN BRC, RBRC-HPS0076

454E2, RIKEN BRC RBRC-HPS0077

### Mice

C.B-17/IcrHsd-*Prkdc^scid^* (SLC Japan)

## Equipment

- Hematocrit capillary tubes (As one, cat. no. 2-454-21)
- 5-ml disposal pipette (As one, cat. no. 1-2247-03)
- 10-ml disposal pipette (As one, cat. no. 1-2247-14)
- Pipetting reservoir (As one, cat. no. 3-5609-03)
- 500-ml bottle-top filter system 0.22-µm (Corning, cat. no. 430769)
- 2-ml disposal pipette (Corning Costar, cat. no.4486)
- 100-mm dishes (Corning Falcon, cat. no. 353003)
- 150-mm dishes (Corning Falcon, cat. no. 351058)
- Pipette controller (Drummond, cat. no. 4-040-135)
- Tweezers for kidney subcapsular transplantation (Dumont, cat. no. 11293-00)
- Multi-channel pipette (Eppendorf, cat. no. 3125000052)
- 15-ml centrifuge tubes (Greiner, cat. no. 188271-013)
- 50-ml centrifuge tubes (Greiner, cat. no. 227261)
- 25-ml disposal pipette (Greiner, cat. no. 760180)
- 50-ml disposal pipette (Greiner, cat. no. 768180)
- 100-mm EZ SPHERE dish (Iwaki, cat. no. 4020-900)
- Inverted fluorescence phase-contrast microscope (Keyence, cat. no. BZ-9000E)
- Refrigerated centrifuge (KUBOTA, cat. no. 6000)
- Cover glasses (Matsunami, cat. no. C024601)
- Glass slides (Matsunami, cat. no. MAS-05)
- Syringe filter 0.22-µm (Millipore, cat. no. SLGVR33RB)
- Small-animal anesthetizer (Muromachi, cat. no. MK-AT210D)
- Needle for hypophysectomy (Natsume Seisakusyo, cat. no. KN-390)
- BICELL (Nihon Freezer)
- 28gauge×13mm needle (NIPRO, cat. no. 08-276)
- Inverted microscope (Olympus, cat. no. CKK41)
- Stereomicroscope (Olympus, cat. no. SZX7)
- CO_2_ incubator (Panasonic Phcbi, cat. no. MCO-19AIC-PJ)
- Tissue-Tek cryomold (Sakura, cat. no. 4566)
- 96-well low-cell-adhesion plate (Sumitomo Bakelite, cat. no. MS-9096V)
- Water bath shaker (TAITEC, cat. no. PERSONAL-11)
- Liquid nitrogen sample storage (Taylor-Wharton, cat. no. 10-033-01-0086)
- Cell counter (Tokyo Garasu Kikai, cat. no. OC-C-S02)
- Cryostat (Thermo Fisher Scientific, cat. no. HM525)
- Cryogenic tubes (Wheaton, cat. no. W985863)

## Reagent setup

### PBS

Dilute 50ml 10× D-PBS (-) with 450ml of deionized water. Autoclave on 30min cycle to sterilize. Store PBS at 4°C for up to 3 months.

### Mitomycin C solution

Reconstitute at 1mg/ml in sterile water.

### DMSO

DMSO is filtered through 0.22-µm syringe filter before use.

### 2-ME

Generate 100mM 2-ME solution by dissolving 25µl 2-ME in 3.5ml PBS. The solution can be stored at 4°C for up to 1 month.

### Collagenase IV solution

Generate 10mg/ml collagenase IV solution by dissolving 0.1mg collagenase IV in 10ml PBS.

### CaCl_2_ solution

Generate 1M CaCl_2_ solution by dissolving 11.1g CaCl_2_ in small amount of water, then adding water to adjust volume to 100ml in total. Aliquot solution (1ml/tube) and store at -30°C.

### Gelatin solution

Generate 0.1% wt/vol gelatin solution by dissolving 0.5g gelatin powder in 500ml deionized water. Autoclave on 30min cycle to dissolve fully and to sterilize. Store gelatin solution at 4°C for up to 3 months.

## bFGF solution

### bFGF high concentration solution

Reconstitute 1mg bFGF with 10ml hPSC maintenance medium to concentration of 100µg/ml. Aliquot solution (100µl/tube) and store at -80°C until use. These aliquots can be stored for up to 6 months.

### bFGF working solution

Retrieve 100µl bFGF high concentration solution from storage at -80°C. Dilute with 20ml hPSC maintenance medium to final concentration of 500ng/ml, aliquot solution (1ml/tube), and store at -30°C until use. These aliquots can be stored for up to 3 months. For use, retrieve 1ml bFGF working solution from storage at -30°C. This solution can be stored at 4°C for up to 4 weeks.

### Dissociation solution

Combine 10ml 10mg/ml collagenase IV solution and 0.25g trypsin powder. Mix well by pipetting to ensure that all residues are dissolved. Add 100µl 1M CaCl_2_ solution and mix further; finally add 30ml PBS. Mix well and sterilize solution by filtering through 0.22-µm syringe filter and add 20ml KSR and 40ml PBS. Mix well and divide solution into aliquots (1.1ml/tube). Store at -30°C until use. These aliquots can be stored for up to 4 months.

### Y-27632 solution

To generate 10mM Y-27632 solution, reconstitute 25mg Y-27632 with 7.3905ml distilled water. Divide solution into aliquots (500µl/tube). This solution can be stored at 4°C for up to two weeks or at -20°C for up to 3 months.

### DNase I

To generate 10mg/ml DNaseI solution, reconstitute 100mg DNase I with 10ml PBS. Divide solution into aliquots (300µl/tube). This solution can be stored at 4°C for up to two weeks or at -30°C for up to 3 months.

### SAG stock solution

To generate 10mM SAG stock solution, reconstitute 10mg SAG with 2.0404ml DMSO. Vortex and spin down. Aliquot solution (100µl/tube) and store at -30°C until use. These aliquots can be stored for up to 3 months.

### SAG working solution

To generate 1mM SAG working solution, dilute 100µl SAG stock solution with 900µl distilled water. This solution can be stored at 4°C (protected from light) for up to one week.

### BMP4 stock solution

To generate 5µM BMP4 stock solution, reconstitute 1mg BMP4 with 5.5ml pre-sterilized 0.1% (wt/vol) BSA/PBS. Aliquot solution (100µl/tube) and store at -30°C until use. These aliquots can be stored for up to 3 months.

### BMP4 working solution

Retrieve 100µl BMP4 stock solution from storage at -30°C. Dilute with 400µl pre-sterilized 0.1% (wt/vol) BSA/PBS to final concentration of 0.5µM. This solution can be stored at 4°C for up to one month.

### Human CRH solution for CRH loading test

100µg CRH are dissolved with 1ml saline. Before use, further dilute to 0.4ug/mL with saline.

## Media for cell culture

### Human PSC maintenance medium

Combine 500ml DMEM/F-12, 125ml KSR, 5ml NEAA, 6.25ml L-glutamine, and 500µl 100mM 2-ME. Sterilize solution by filtering through 0.22-µm bottle-top filter, store at 4°C, and use within a month. Basic FGF should be freshly added to culture medium at each medium change. For 10ml maintenance medium, add 100µl bFGF working solution so that final concentration of bFGF is 5ng/ml.

### MEF medium

DMEM with 10% (vol/vol) FBS. Sterilize solution by filtering through 0.22-µm bottle-top filter. Store at 4°C for up to 1 month.

### Growth factor-free chemically defined medium (gfCDM)

Prepare by combining 250ml IMDM (containing GlutaMAX), 250ml F-12 (containing GlutaMAX), 5ml CDLC, 10ml 250mg/ml BSA, and 19.5µl 1-thioglycerol. Sterilize solution by filtering through 0.22-µm bottle-top filter, store at 4°C, and use within 2 weeks.

### Pituitary differentiation medium (gfCDM supplemented with 10% and 20% KSR)

Prepare gfCDM supplemented with 10% and 20% (vol/vol) KSR. Mix gently using pipette and sterilize solution by filtering through 0.22-µm bottle-top filter, store at 4°C, and use within 2 weeks.

## Preparation of frozen stock of MEF feeder cells

### 1 Thawing MEF feeder cells

A) Put 10ml MEF medium into 15-ml centrifuge tube and 50ml MEF medium into 50-ml centrifuge tube. Pre-warm tubes in 37°C water bath.
B) Transfer one frozen tube containing MEF feeder cells from liquid nitrogen.
C) Half-thaw MEF feeder cells in 37°C water bath.
D) Take about 1ml MEF medium from 15-ml centrifuge tube in A), add to tube in C), thaw MEF cells completely, and quickly collect cells in 15-ml centrifuge tube in A).
E) Centrifuge MEF feeder cells at 170g for 5 min, aspirate supernatant, and resuspend MEF feeder cells in 5ml MEF medium from 50-ml centrifuge tube in A).
F) Add 9ml MEF medium from 50-ml centrifuge tube in A) to each of five 100-mm cell culture dishes.
G) Seed 1 ml cell suspension in E) into each 100-mm cell culture dish in F).
H) Incubate dishes at 37°C in 5% CO_2_ incubator.
I) On the next day, replace medium with 10ml/dish of pre-warmed MEF medium.

### 2 Passage MEF feeder cells

A) Pre-warm 465ml MEF medium, 50ml PBS, and 10ml 0.05% trypsin-EDTA in 37°C water bath.
B) Aspirate culture medium from confluent five MEF dishes and wash dish contents with 10ml PBS.
C) Add 2ml/dish 0.05% trypsin-EDTA.
D) Incubate dishes at 37°C in 5% CO_2_ incubator for 5 min.
E) Remove MEF feeder cells by tapping dishes and pipetting cells to generate single-cell suspension.
F) Collect cell suspension in 50-ml centrifuge tube.
G) With 3 ml MEF medium, collect remaining cells in 50-ml centrifuge tube in F).
H) Collect remaining cells from 5 dishes with 10ml MEF medium once again.
I) Centrifuge at 170g for 5 min, aspirate supernatant, and resuspend pellet in 5ml MEF medium.
J) Add 25ml MEF medium to I) and suspend well.
K) Add 28ml pre-warmed MEF medium in A) to each of fifteen 150-mm cell culture dishes.
L) Seed 2 ml cell suspension in J) into each 150-mm cell culture dish in K).
M) Incubate dishes at 37°C in 5% CO_2_ incubator.

### 3 Treatment with mitomycin C

A) Pre-warm 450ml MEF medium and 1000ml DMEM in 37°C water bath.

B) Add 300µl 1mg/ml mitomycin C solution to each confluent MEF 150-mm dish (30ml MEF medium/dish). Final concentration of mitomycin C is 10μg/ml.

C) Incubate dishes at 37°C in 5% CO_2_ incubator for 3 hours.

D) Remove MEF medium containing mitomycin C by aspiration.

E) Wash 150-mm dishes three times with 22ml DMEM/dish.

F) Add 25ml MEF medium and incubate overnight at 37°C in 5% CO_2_ incubator.

### 4 Cryopreservation of MEF feeder cells

A) BICELL should be chilled to 4°C.
B) Mix 1ml filter-sterilized DMSO with 9ml MEF medium and place on ice.
C) Pre-warm 75ml MEF medium and 500ml PBS in 37°C water bath.
D) Perform following treatments in triplicate, 5 plates at a time: Aspirate culture medium and rinse with 30ml PBS/150-mm dish.
E) Add 5ml 0.05% trypsin-EDTA/150-mm dish.
F) Incubate dishes at 37°C in 5% CO_2_ incubator for 5 min.
G) Remove MEF feeder cells by tapping dishes and pipetting to generate single-cell suspension.
H) Collect cell suspension in 50-ml centrifuge tube.
I) Add 5ml MEF medium/150-mm dish and collect remaining cells.
J) After mixing well, take 50µl for cell count.
K) Centrifuge cell suspension in H) at 170g for 5 min and aspirate supernatant.
L) Resuspend to 6.0 × 10^6^ cells/ml pre-cooled MEF medium with DMSO in B).
M) Seed MEF feeder cells for hPSCs maintenance at 1.2 × 10^6^ MEF feeder cells/100-mm dish. Cells thus should be stocked in multiples of 1.2 × 10^6^ cells. For example, we label cryogenic tube lids as 1.2 × 10^6^ cells→M12, 2.4 × 10^6^ cells→M24, 3.6 × 10^6^ cells→M36, or 4.8 × 10^6^ cells→M48.
N) Place cryogenic tubes into pre-cooled BICELL in A) and transfer to -80°C overnight.
O) On next day transfer cryogenic tubes into liquid nitrogen.

## Maintenance of hPSCs on MEF feeder cells

### Seeding of hPSCs for one 100-mm dish is described

#### 1 Plating MEF feeder cells

A) Coat 100-mm dish with 8ml 0.1% (wt/vol) gelatin solution and allow plate to rest at room temperature for 15 min.
B) Aspirate gelatin solution from 100-mm dish using Pasteur pipette and add mitomycin C-treated MEF suspension in MEF medium (10ml/100-mm dish). Optimal MEF cell numbers: 1.2 × 10^6^ cells/100-mm dish.
To seed MEF feeder cells at appropriate density is important to avoid hPSC differentiation. MEFs seeded at this density can usually be used for hPSC culture 1 or 2 days after seeding.
C) Incubate MEF feeder cells at 37°C in 5% CO2 incubator overnight.

#### 2 Thawing hPSCs on MEF feeder cells

A) Prepare two 15-ml centrifuge tubes containing 10ml hPSC maintenance medium. Pre-warm them in 37°C water bath.
B) Remove a cryopreserved cell vial from liquid nitrogen.
C) Add about 1ml medium warmed in A) to vial and quickly thaw cells by pipetting gently.
D) Transfer cell suspension to 15-ml centrifuge tube in A).
E) Centrifuge at 170g for 5 min and aspirate as much supernatant as possible.
F) Prepare 10ml new human PSC maintenance medium containing 100µl bFGF working solution (final concentration bFGF: 5ng/ml). Add 1ml to E) and suspend cells gently. Transfer suspension to remaining 9ml medium.
G) Remove MEF medium from dish of MEF feeder cells.
H) Seed 10ml cell suspension in F) onto MEF feeder cells in G).
I) Incubate dish at 37°C in 2% CO_2_ incubator.
J) Human PSCs must be fed daily by replacing old medium with 10ml new hPSC maintenance medium with 100µl bFGF working solution.
K) After 2 or 3 days, check hPSC appearance.
  Healthy hPSCs form flat colonies with high cell density centrally and sharp colony edges. Do not let hPSCs overgrow: That results in differentiation and cell death (**Figures 2B, C**).

#### 3 Passage of hPSCs

A) Prepare required number of MEF feeder cell 100-mm dishes (3–6 dishes) on day before passage. The procedure is as in 1. Plating MEF feeder cells.
B) Prepare two 15-ml centrifuge tubes containing 10ml hPSC maintenance medium and one or two 50-ml centrifuge tubes containing 30ml hPSC maintenance medium.
Pre-warm them in 37°C water bath.
C) Aspirate all hPSC maintenance medium and quickly wash hPSCs with 10ml PBS. Add 1.0ml dissociation solution, ensuring that solution covers all cells.
D) Incubate hPSCs at 37°C in 2% CO_2_ for 5–8 min.
E) Place under microscope (×4 objective lens).
Observe colonies to confirm that edges are starting to detach.
F) Tap plate 3–4 times to facilitate cell detachment. Add 5ml pre-warmed hPSC maintenance medium and transfer cell suspension to new 15-ml centrifuge tube (★). To collect cells remaining in dish, add another 5ml pre-warmed hPSC maintenance medium and transfer cell suspension into same 15-ml centrifuge tube (★).
G) Centrifuge 15-ml centrifuge tube at 170g for 1 min at room air.
H) Aspirate supernatant and resuspend pellet in 1ml hPSC maintenance medium.
I) Very gently pipette cell pellet into small fragments (about 100–200 µm) using P1000 tip. Resuspend each pellet in 30–50 ml of hPSC maintenance medium containing 300µl–500µl bFGF working solution. The split ratio is 1:3–1:6.
J) Remove medium from pre-seeded MEF dishes.
K) Plate 10ml cell suspension in I) into each 100-mm MEF feeder dish.
L) Incubate dishes at 37°C in 2% CO_2_ incubator.

## Differentiation of pituitary corticotroph cells from hPSCs.

### Day 0

#### 1 Deplete MEF feeder cells

A) Prepare two 15-ml centrifuge tubes containing 10ml PBS and two 15-ml centrifuge tubes containing 10ml hPSC maintenance medium. Pre-warm them in 37°C water bath.
B) Coat 100-mm dish with 8ml 0.1% (wt/vol) gelatin solution and allow plate to rest at room temperature for 15 min.
C) Aspirate all hPSC maintenance medium from dish containing confluent hPSCs. Wash dish with 10ml pre-warmed PBS. Remove PBS. Add 1.0ml dissociation solution, ensuring that solution covers all cells.
D) Incubate cells at 37°C in 2% CO_2_ for 8 min.
E) Observe colonies by microscope to confirm that edges of hPSCs are starting to detach.
F) Tap dish to facilitate cell detachment. Add 5ml pre-warmed hPSC maintenance medium containing 2µl DNase I and transfer detached cell suspension to 15-ml centrifuge tube. To collect cells remaining in dish, add another 5ml of same medium and transfer cell suspension to 15-ml centrifuge tube.
G) Centrifuge 15-ml tube at 170g at room temperature for 1 min.
H) Remove supernatant and resuspend pellet in 10ml pre-warmed hPSC maintenance medium with 10 µM Y-27632.
I) Aspirate gelatin solution from dish set up in B).
J) Seed cell suspension in H) into gelatin-coated dish.
K) Incubate dish at 37°C in 2% CO_2_ incubator for 90 min, allowing MEF feeder cells to adhere to bottom of dish. To minimize numbers of MEF feeder cells brought in is desirable for pituitary differentiation.

#### 2 Serum-free floating culture of embryoid body-like aggregates with quick reaggregation (SFEBq)

A) Prepare 15-ml centrifuge tube containing 10ml PBS, 15-ml centrifuge tube containing 10ml gfCDM, and 15-ml centrifuge tube containing 13ml gfCDM + 10% KSR. Pre-warm them in 37°C water bath.
B) Pre-wet 10-ml disposal pipette with hPSC maintenance medium to avoid cell adhesion to pipette wall. Gently transfer detached fragment of hPSC colonies
into a fourth 15-ml centrifuge tube.
C) Centrifuge at 170g for 10 sec.
D) Remove supernatant and resuspend pellet gently in 10ml pre-warmed PBS.
E) Centrifuge at 170g for 10 sec.
F) Remove supernatant and resuspend pellet in 1.0ml–1.5ml of TrypLE Express containing 67 µg/ml DNase I and 67 µM Y-27632.
G) Incubate in 37°C water bath for 2–3 min.
H) Dissociate pellet very gently into single cells using P1000 tip and resuspend them in 10ml pre-warmed gfCDM.
This step is the most difficult step in the protocol. Pipetting technique and exposure time to TrypLE must be adjusted not to damage hPSCs. To avoid cell death, make sure pipette tip does not rest against centrifuge-tube bottom.
I) Centrifuge at 170g for 3 min.
J) Remove supernatant and resuspend cells very gently in 1ml pre-warmed gfCDM + 10% KSR containing 20µM Y-27632.
K) Count dissociated hPSCs using a cell counter.
L) Adjust cell density to 1 × 10^5^ cells/ml with pre-warmed gfCDM + 10% KSR containing 20µM Y-27632.
M) Plate hPSCs into V-bottomed 96-well low-cell-adhesion plate (10,000 cells/100µl well) using multi-channel pipette and pipetting reservoir.
To prevent desiccation, add 200µl PBS/well to outer 36 wells of 96-well plate and seed hPSCs into inner 60 wells.
N) Incubate plate at 37°C in 5% CO_2_ incubator.

#### 3 Differentiation into early pituitary primordium in 3D culture of hPSCs

### Days 3–30

New medium should be pre-warmed in 37°C water bath before medium change.

**Figure 3:**
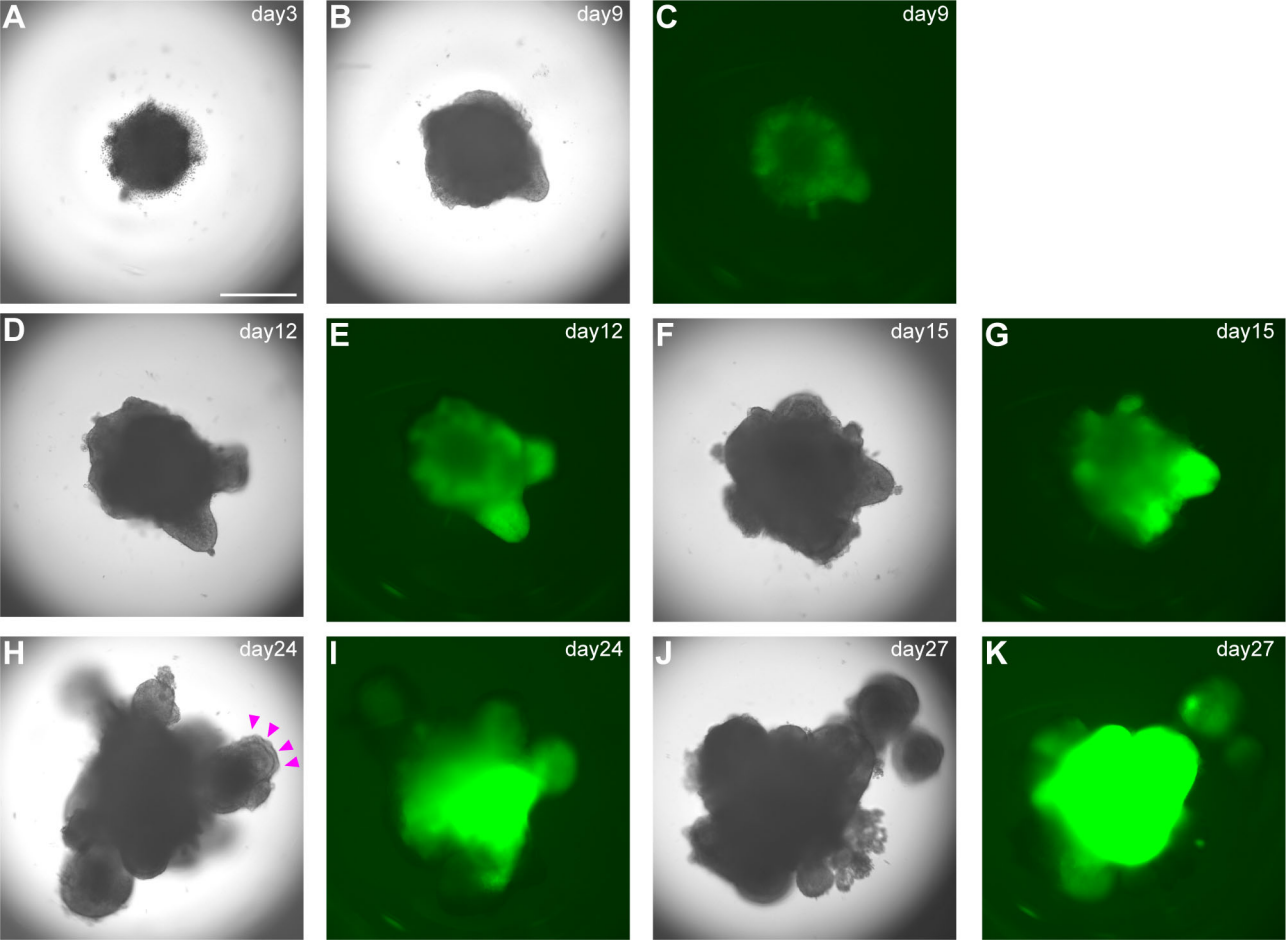
Differentiation into early pituitary primordium in SFEBq culture. **(A–K)** Bright-field and EGFP fluorescence microscopy views of aggregates in SFEBq culture. We used the KhES-1_Rx::Venus (VA22-N37) cell line, carrying Venus knocked into the Rx locus (13). RX::Venus-expressing hypothalamic tissues are visible from day 9. On day 24, oral ectoderm-like tissue appears on the surface of the aggregate (**H**, magenta arrowheads). This feature can be clearly identified in bright-field. Scale bar: 500 μm.

A) Day 3: Check by microscopy (we routinely use inverted fluorescence phase-contrast microscope BZ-9000) if hPSC sphere-shaped aggregates have formed. On day 3, healthy aggregates reach 400–500 µm in diameter without dead cells (**Figure 3A**). Add gfCDM + 10% KSR (100 µl/well) without Y-27632 to each well. Medium should be added gently to avoid disturbing aggregates.
B) Day 6: Add SAG and BMP4 to pituitary differentiation medium. Remove half of old medium (100µl/well) and add same volume (100µl/well) of gfCDM + 10% KSR with 4µM SAG and 20 nM recombinant human BMP4. Final concentrations of SAG and BMP4 are 2µM and 10nM respectively.
  Medium should be removed and added gently. Do not disturb aggregates.
C) Day 9, 12, and 15: Medium change. Change the medium by removing half of old medium and replacing with same volume gfCDM + 10% KSR supplemented with 2µM SAG and 10nM BMP4 (**Figures 3C–G**).
D) Day 18, 21, and 24: Medium change. After day 18 content of BMP4 in pituitary differentiation medium is gradually reduced. Change medium by removing half of old medium and replacing it with same volume of gfCDM + 10% KSR supplemented with 2µM SAG (without BMP4) (**Figures 3H–K**). Incubate plate under 5% CO_2_ and 40% O_2_. On days 18–24, formation of multiple cysts indicates failure of differentiation into pituitary gland. Re-start from SFEBq.
E) Day 30: Transfer. Transfer aggregates from 96-well plate into 100-mm EZ SPHERE dish (density ~60 aggregates/dish) containing 13ml gfCDM + 10% KSR supplemented with 2µM SAG. Start to incubate EZ SPHERE dish at 37°C under 5% CO_2_ and 40% O_2_. Before transfer, wash aggregates with gfCDM to remove BMP4 and to end its signals.

#### 4 Differentiation of hPSC-derived pituitary progenitors into ACTH-producing tissues

New medium should be warmed before medium change.

### Days 31–50

Medium change: From day 30, change medium every third day by replacing old medium with 10ml gfCDM + 10% KSR supplemented with 2µM SAG. Culture at 37°C under 5% CO_2_ and 40% O_2_.

### Days 51–360 or more

Increase concentration of KSR in medium from 10% to 20% after day 50. Change medium every third day by replacing old medium with 10ml gfCDM + 20% KSR supplemented with 2µM SAG. Culture at 37°C under 5% CO_2_ and 40% O_2_. These aggregates can be cultured for more than one year. Long-term cultures increase risk of contamination. Check color and odor of culture medium when changing medium.

### Expected results

Around day 18, you can visually identify superficial layers composed of pituitary progenitor cells around the organoids in bright field (**Figure 3H**). These superficial tissues rarely surround the entire circumference of the organoid and are usually partial. Furthermore, each differentiation stage of pituitary organoids can be evaluated by immunostaining. We observe organoid appearance on inverted fluorescence phase-contrast microscopy until day 30 and thereafter by immunostaining on days 30, 40, 60, and 100. Aggregates are fixed in 4% paraformaldehyde for 10 min at 4°C. Fixative is replaced by incubation in 20% sucrose overnight at 4°C. Aggregates are mounted in OCT embedding compound and frozen at -80°C. Frozen tissues are sectioned at 10µm. Cryosections are picked up on glass slides, washed in 0.3% Triton X-100/PBS for permeabilization, and washed with PBS three times. Sections are incubated in 2% (w/v) dry skimmed milk/PBS for 60 min at room temperature (RT) for blocking. Sections are incubated overnight at 4°C with primary antibodies diluted in 2% dry skimmed milk/PBS. Sections are then washed in 0.05% Tween 20/PBS and incubated with secondary antibodies diluted in 2% dry skimmed milk/PBS for 2 hours at RT. DAPI is added to mark cell nuclei.

### Day 30

**Figure 4:**
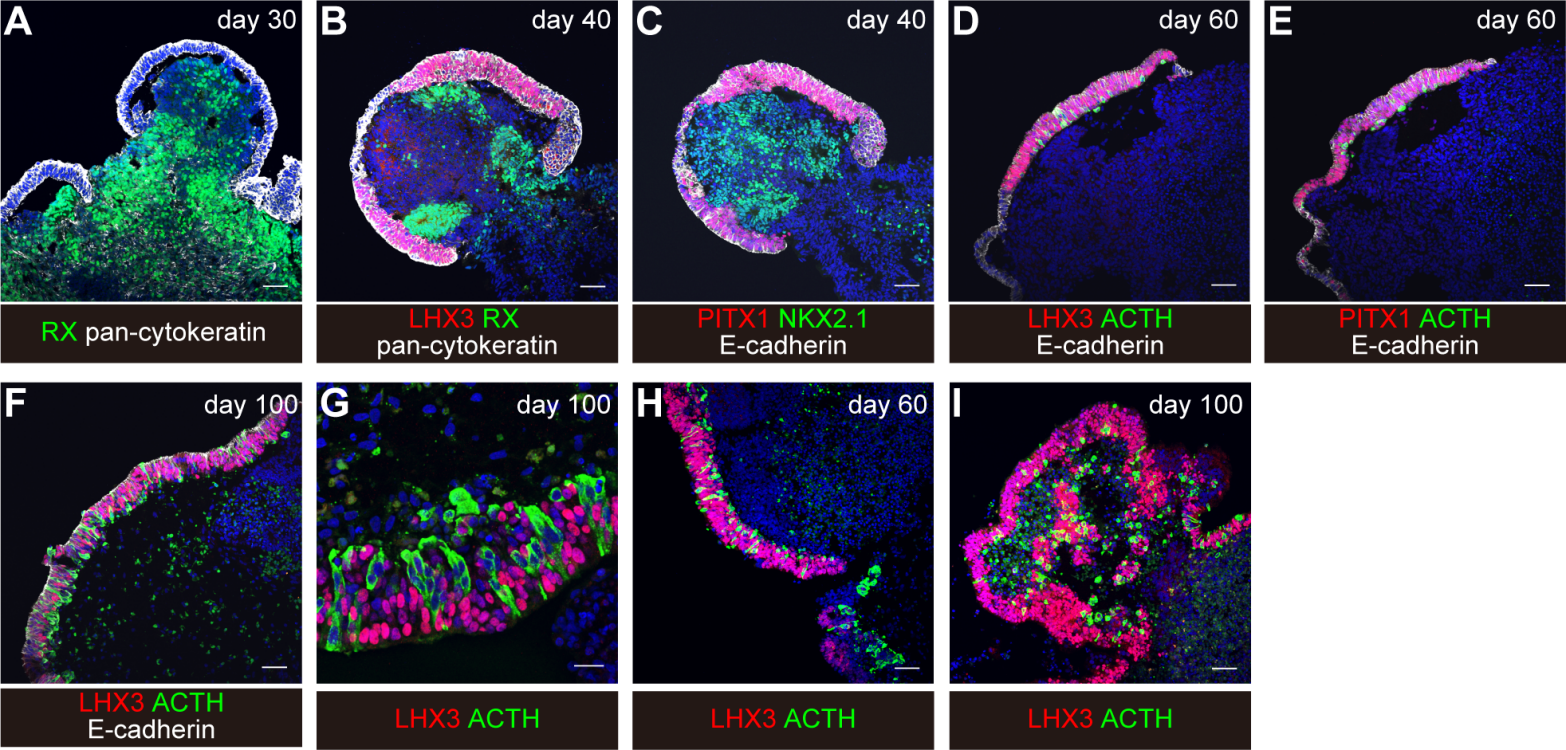
Induction of differentiation of human iPSCs into corticotroph cells. From “Hypothalamic contribution to pituitary functions is recapitulated *in vitro* using 3D-cultured human iPS cells,” by T. Kasai and H. Suga, 2020, Cell Reports 30, 18-24. e15. https://doi.org/10.1016/j.celrep.2019.12.009. Creative Commons CC-BY license. **(A–G)** original condition. **(H, I)** modified condition. Scale bars: 50μm **(A–F, H, I)** and 20μm **(G)**.

Focal surface expression of oral ectoderm marker pan-cytokeratin and of PITX1 is seen (**Figure 4A**).

### Day 40

Thickened pan-cytokeratin- and PITX1-expressing oral ectoderm expresses pituitary progenitor marker LHX3 and E-cadherin (**Figures 4B, C**). The interior hypothalamic precursor-like tissue expresses RX and Nkx2.1. Superficial ectoderm cells in small numbers express ACTH.

### Day 60–100

With continuous culture, more ACTH-expressing cells are observed (**Figures 4D–I**).

On day 60, about 30% of the aggregates contained both LHX3^+^ cells and ACTH^+^ cells. About 60% of the aggregates contained LHX3^+^ pituitary precursor cells.

#### 5 Functional assessment of hPSC-derived ACTH-producing tissues *in vitro*

### Basal ACTH measurement *in vitro*

To evaluate basal secretion of ACTH from hESC-derived pituitary ACTH concentrations in conditioned medium are measured. Full medium change (10ml/30 aggregates) is performed and culture supernatant is collected after 24 hours of culture at 37°C under 5% CO_2_ and 40% O_2_. Culture supernatant (200µl) collected in 1.5ml-tube is immediately frozen at -150°C. ACTH concentration is determined by electrochemiluminescence immunoassay (ECLIA) kit, which is in widespread clinical use in Japan.

### Expected Results

Baseline ACTH concentration in culture medium without any aggregates (negative control) is 4.8 ± 0.12pg/ml. ACTH concentrations in aggregate-containing medium are 29.6 ± 8.14pg/ml on day 60 and 289.19 ± 67.362pg/ml on day 100 (8).

### CRH loading test *in vitro*

On days 113–152, eight to sixteen aggregates are used for CRH loading test. Full medium change is performed with gfCDM + 20% KSR + 2mM SAG and culture supernatant is collected after 24 hours of culture at 37°C under 5% CO_2_ and 40% O_2_. Another full medium change is performed with gfCDM + 20% KSR + 2mM SAG + human CRH (5mg/ml) and culture supernatant is again collected after 24 hours of culture at 37°C under 5% CO_2_ and 40% O_2_. ACTH concentrations in supernatants before and after CRH addition are determined.

### Expected results

On days 113–152, ACTH concentrations are approximately twofold increased, from 776 ± 130.67pg/ml to 1060 ± 302.02pg/ml after CRH stimulation.

#### 6 Functional assessment of hPSC-derived ACTH-producing tissues *in vivo*

### Hypophysectomy of SCID mice

**Figure 5:**
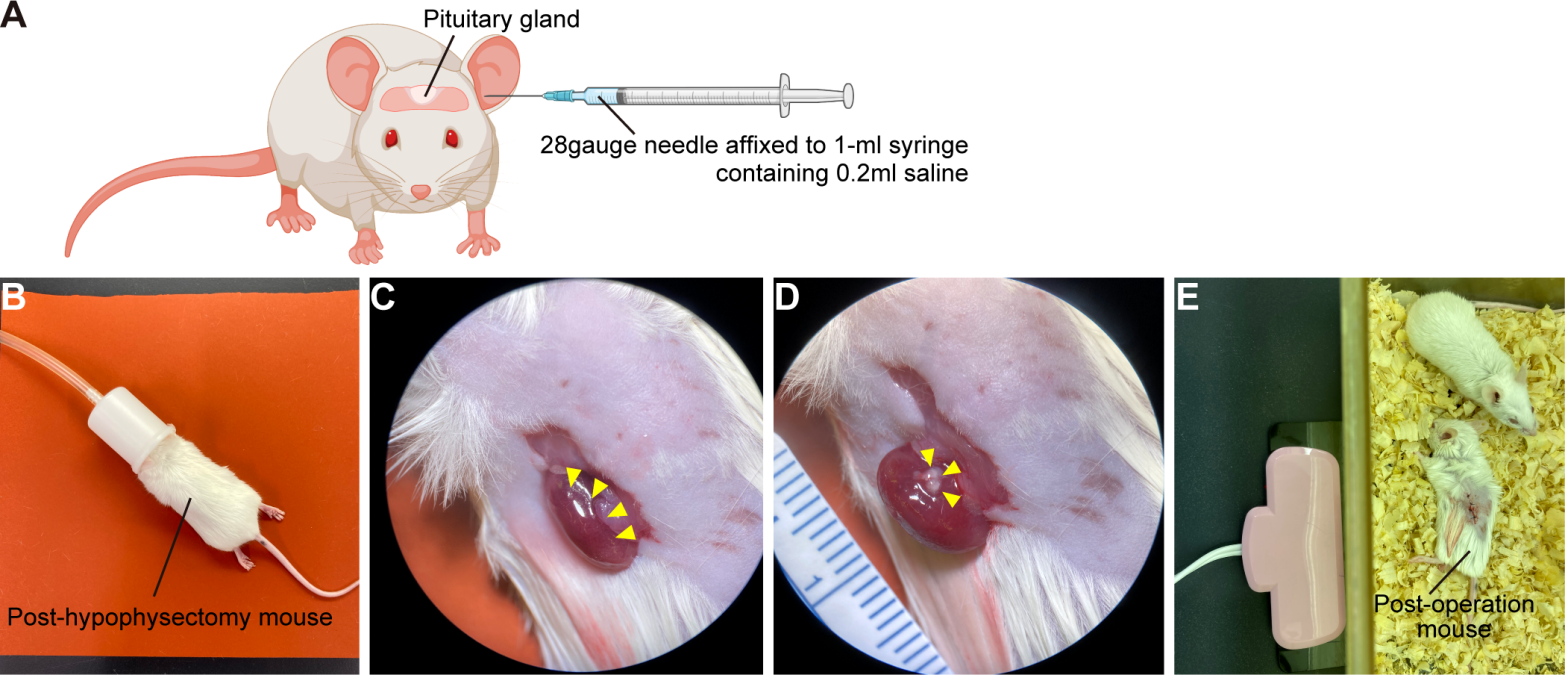
Kidney subcapsular transplantation of the hPSC-derived pituitary tissue into post-hypophysectomy mice. **(A)** Hypophysectomy of SCID mice. Created with BioRender (https://biorender.com). **(B)** Post-hypophysectomy mice are anaesthetized with isoflurane. Before the operation, mice are injected with 0.2mg dexamethasone and 2mg ampicillin intramuscularly. **(C)** Exposure of a kidney (yellow arrowheads). **(D)** The hPSC-derived pituitary tissue (yellow arrowheads) is transplanted under the renal capsule. **(E)** Post-transplantation mouse. Mice must be kept warm until they fully awaken from anesthesia.

A) Eight-week-old SCID mice are anaesthetized with intraperitoneal injection of a mixture of three anesthetics (medetomidine 0.75mg/kg, midazolam 4mg/kg, butorphanol 5mg/kg). Because mixed anesthesia induces hypothermia, mice must be maintained at 37°C using an animal warmer.
B) Prepare 28gauge needle affixed to 1-ml syringe containing 0.2ml saline.
C) If surgeon is right-handed: Holding mouse head in left hand and needle in right, insert needle along left external auditory canal.
D) Pass needle through left eardrum. (**Figure 5A**)
E) Pass needle tip into interosseous space and beyond to pituitary gland.
F) With right thumb, aspirate 100µl–300µl of material – importantly, without moving needle tip. Confirm aspiration of pituitary gland into syringe.
G) Withdraw syringe and needle without further manipulation.
H) If there is bleeding from the ear, stop by pressing down with swab.
I) Administer atipamezole, 3mg/kg, intraperitoneally. Mice must be kept warm.
J) After checking that mice are fully awake, return them to cages.
K) Seven days after hypophysectomy, CRH loading test is performed to confirm hypopituitarism.
L) Mice subjected to hypophysectomy mice are anaesthetized with isoflurane and CRH, 2µg/kg, is injected intraperitoneally. EDTA plasma samples are obtained before start of injection and 30 min thereafter. Plasma ACTH concentrations are determined using ACTH ELISA kit.

### Expected results

Hypopituitarism is present when CRH-stimulated plasma ACTH levels are less than 10pg/ml.

### Kidney subcapsular transplantation

A) Under stereomicroscopy, detach only superficial pituitary tissue from pituitary organoids with tapered tweezers.
B) Post-hypophysectomy mice are anaesthetized with isoflurane (**Figure 5B**).
C) Before surgery, mice are injected with dexamethasone (0.2mg) and ampicillin (2mg) intramuscularly to prevent adrenal crisis and infection.
D) Lay mouse on its side and finger-palpate kidney position.
E) Shave surgical field.
F) Incise flank tissues to expose kidney (**Figure 5C**). Handle kidney gently. In particular, bleeding from renal artery at origin of renal pelvis can be fatal.
G) Under stereomicroscopy, push hPSC-derived pituitary tissue under renal capsule with tapered tweezers.
H) Confirm that pituitary tissue lies beneath renal capsule (**Figure 5D**) and close wound.
I) Mice must be kept warm until they fully awaken from anesthesia (**Figure 5E**).

### Expected results

CRH loading test response is restored 10 days after transplantation (7).

## Discussion

Pituitary organoids derived from hPSCs offer promise for regenerative medicine. However, some challenges remain to be overcome to realize this goal. First, pituitary organoids must be generated under Current Good Manufacturing Practice conditions to permit fully safe transplantation and human PSCs must be maintained without MEF feeder cells to avoid xeno-contamination. In monolayer culture the proportion of ACTH-expressing cells was <20% on immunofluorescence analysis (10). Using our method 30% of the organoids contained both LHX3-expressing cells and ACTH-expressing cells. Differentiation efficiency thus must be improved, taking multiple factors into consideration, including culture medium and signaling molecules. While both SAG and BMP4 appear essential for pituitary differentiation from human PSCs (7–10), other signaling molecules are involved in pituitary development. Wnt signaling plays an essential role in anterior–posterior neural patterning (14). The most anterior cranial placode, the origin of the adenohypophysis, requires Wnt inhibition while the most posterior placode depends on active Wnt signaling. In 2D induction, Wnt inhibition rather than high BMP levels are important for the transition from surface ectoderm to early placode (9).

Our pituitary organoids secrete anterior pituitary hormones such as GH (7) and PRL (15). Since supply of multiple anterior pituitary hormones is impaired in panhypopituitarism, the induction efficiency of multiple lineages should be improved.Pituitary organoids derived from hPSCs demonstratedly can function as a transplanted organ when ectopically transplanted into immunodeficient mice. In preclinical work POC experiments using non-human primates or large animals such as pigs may be required. A hypophyseal dysfunction model in cynomolgus monkey is available (16), permitting investigations of transplantation procedure, suitable transplant location (orthotopic or ectopic), and graft endocrinologic function. Xenotransplantation of human PSC-derived pituitary tissue into monkeys with hypopituitarism of course requires immunosuppression. This situation differs from that to be addressed by autologous transplantation (patient’s own PSC-derived pituitary→patient). Care will thus be required when interpreting experimental results obtained from human-monkey transplantation. Finally, neural stem/progenitor cells are present throughout the inner hypothalamic tissues in pituitary organoids. These neural precursor cells could form tumors after transplanatation. Isolation and transplantation of pure corticotroph cells sufficiently matured in pituitary organoids thus may be required for quality control. Despite some challenges, pituitary organoids potentially may provide novel treatment for patients suffering from hypopituitarism, overcoming the disadvantages of current therapies.

Last, clinical applications are not the only use for pituitary organoids. Pituitary organoids can be deployed in basic research that would be impossible in humans for ethical or safety reasons. They will contribute to endocrinology by permitting the discovery of mechanisms of hypothalamus-pituitary development and of hereditary hypopituitarism.

## Data availability statement

The original contributions presented in the study are included in the article/supplementary material. Further inquiries can be directed to the corresponding authors.

## Ethics statement

The animal study was reviewed and approved by Nagoya University Graduate School of Medicine.

## Author contributions

MK and HSu conceived the idea. MK wrote the manuscript. HSu revised the manuscript. HSa and TM contributed to data preparation. All authors contributed to the article and approved the submitted version.
